# Supplementary material for: Women’s perception about the discovery of breast cancer amid the covid-19 pandemic
Source: PLoS One. 2023 Apr 27;18(4):e0282610. doi: 10.1371/journal.pone.0282610 (PMC10138248; doi:10.1371/journal.pone.0282610)
Supplement: Attachment — Submitted filename: Response to Reviewers.docx [file pone.0282610.s001.docx]

PONE-D-22-24658

Women’s perception about the discovery of breast cancer in the middle of the covid-19 pandemic

PLOS ONE

Dear Dr. Carvalho,

Thank you for submitting your manuscript to PLOS ONE. After careful consideration, we feel that it has merit but does not fully meet PLOS ONE’s publication criteria as it currently stands. Therefore, we invite you to submit a revised version of the manuscript that addresses the points raised during the review process.

- Author(s) need to be specific on the methodology they intend to use to avoid confusing readers. For example, the author(s) uses qualitative methods of data collection (interview) but end-up using the tools (SPSS) for quantitative methods of data analysis.
- Reviewers differ in decision because of author(s) methodological flaw. To seek for statistical significant you may need to use questionnaire with larger population, although combining methods might enhance the work. If you have available data for such mixed methods, then it will be fantastic to combine both.
- Author(s) can report part of qualitative data using table(s), but sophisticated analytical tools for qualitative data cannot be utilized to generate such result.

Please submit your revised manuscript by Jan 09 2023 11:59PM. If you will need more time than this to complete your revisions, please reply to this message or contact the journal office at [plosone@plos.org](mailto:plosone@plos.org). When you're ready to submit your revision, log on to https://www.editorialmanager.com/pone/ and select the 'Submissions Needing Revision' folder to locate your manuscript file.

Please include the following items when submitting your revised manuscript:

- A rebuttal letter that responds to each point raised by the academic editor and reviewer(s). You should upload this letter as a separate file labeled 'Response to Reviewers'.
- A marked-up copy of your manuscript that highlights changes made to the original version. You should upload this as a separate file labeled 'Revised Manuscript with Track Changes'.
- An unmarked version of your revised paper without tracked changes. You should upload this as a separate file labeled 'Manuscript'.

If you would like to make changes to your financial disclosure, please include your updated statement in your cover letter. Guidelines for resubmitting your figure files are available below the reviewer comments at the end of this letter.

If applicable, we recommend that you deposit your laboratory protocols in protocols.io to enhance the reproducibility of your results. Protocols.io assigns your protocol its own identifier (DOI) so that it can be cited independently in the future. For instructions see: <https://journals.plos.org/plosone/s/submission-guidelines#loc-laboratory-protocols>. Additionally, PLOS ONE offers an option for publishing peer-reviewed Lab Protocol articles, which describe protocols hosted on protocols.io. Read more information on sharing protocols at <https://plos.org/protocols?utm_medium=editorial-email&utm_source=authorletters&utm_campaign=protocols>.

We look forward to receiving your revised manuscript.

Kind regards,

Adetayo Olorunlana, Ph.D.
Academic Editor
PLOS ONE

Journal Requirements:

When submitting your revision, we need you to address these additional requirements.

1. Please ensure that your manuscript meets PLOS ONE's style requirements, including those for file naming. The PLOS ONE style templates can be found at

https://journals.plos.org/plosone/s/file?id=wjVg/PLOSOne_formatting_sample_main_body.pdf and

<https://journals.plos.org/plosone/s/file?id=ba62/PLOSOne_formatting_sample_title_authors_affiliations.pdf>

2. Please provide additional details regarding participant consent. In the ethics statement in the Methods and online submission information, please ensure that you have specified (1) whether consent was informed and (2) what type you obtained (for instance, written or verbal, and if verbal, how it was documented and witnessed). If your study included minors, state whether you obtained consent from parents or guardians. If the need for consent was waived by the ethics committee, please include this information.

**Answer**: The study was carried out upon the receipt of the Free and Informed Consent Form, in written format, signed by all those who agreed to participate in the study (inserted in the Article).

If you are reporting a retrospective study of medical records or archived samples, please ensure that you have discussed whether all data were fully anonymized before you accessed them and/or whether the IRB or ethics committee waived the requirement for informed consent. If patients provided informed written consent to have data from their medical records used in research, please include this information.

3. Thank you for stating the following financial disclosure:

“No”

At this time, please address the following queries:

a)        Please clarify the sources of funding (financial or material support) for your study. List the grants or organizations that supported your study, including funding received from your institution.

**Answer**: There was no funding of any kind. (inserted in the Article)

b)        State what role the funders took in the study. If the funders had no role in your study, please state: “The funders had no role in study design, data collection and analysis, decision to publish, or preparation of the manuscript.”

c)        If any authors received a salary from any of your funders, please state which authors and which funders.

d)        If you did not receive any funding for this study, please state: “The authors received no specific funding for this work.”

**Answer**: There was no funding of any kind; all costs, both from research and from the submission of the article, were subsidized by the main researcher. (inserted in the Article)

Please include your amended statements within your cover letter; we will change the online submission form on your behalf.

4. We note that you have stated that you will provide repository information for your data at acceptance. Should your manuscript be accepted for publication, we will hold it until you provide the relevant accession numbers or DOIs necessary to access your data. If you wish to make changes to your Data Availability statement, please describe these changes in your cover letter and we will update your Data Availability statement to reflect the information you provide.

**Answer**: data will not be made available.

Considering that part of the interviews contain information that might identify the participants, the researchers committed themselves to treat the identity of the participants according to professional standards of confidentiality, since they contain particular elements and names of people and/or professionals. Thus, the investigation was based on the Brazilian norms that regulate research with human beings (resolution no. 466, of December 12, 2012, of the National Health Council/Ministry of Health) that uphold the confidentiality of the identification data of the research members, we chose not to provide the complete transcripts. However, speech clippings (transcriptions) were used as a way to represent the findings of the study, seeking to exemplify the categories listed from the content analysis applied.

[Note: HTML markup is below. Please do not edit.]

Reviewers' comments:

Reviewer's Responses to Questions

**Comments to the Author**

1. Is the manuscript technically sound, and do the data support the conclusions?

The manuscript must describe a technically sound piece of scientific research with data that supports the conclusions. Experiments must have been conducted rigorously, with appropriate controls, replication, and sample sizes. The conclusions must be drawn appropriately based on the data presented.

Reviewer #1: No

Reviewer #2: No

Reviewer #3: Yes

**Answer 1.1**: Initially, we the authors would like to thank the observations, questions and suggestions made by the editor and by each reviewer. The scores collaborate with the improvement of this work and enrich the learning for future research and academic productions, contributing with other perspectives to the focus of the research. Thus, we hope to have implemented all the modifications and have complied with every suggestion or request made.

**Answer 1.2**: This research reflects a cross-sectional approach to a larger study, which is in progress. The proposal was to present a descriptive, exploratory study design with a strictly qualitative approach. Regarding the number of participants, the study had 40 interviews that, based on other studies (see "strengths and limitation of the study"), are sufficient to understand the event studied, that is, the perception of women about the discovery of breast cancer. However, we understand that it is not possible to generalize the findings to other samples, and it is not our intent to be generalizable. In this sense, the conclusions of the research aim to shed some light on the need for an educational approach to identify signs and symptoms that favor screening and early diagnosis of breast cancer. Furthermore, we wish to highlight the importance of preparing the professional team to consider and welcome the emotional impact generated by the diagnosis of a stigmatized disease, considering the support network that women need to cope with the situation.

2. Has the statistical analysis been performed appropriately and rigorously?

Reviewer #1: No

Reviewer #2: No

Reviewer #3: Yes

**Answer 1**: This study is strictly qualitative, so no statistical analysis was performed. Only sociodemographic and clinical data were presented in absolute and relative frequency format. The presentation was corrected in the article, to avoid confusion to the reader. (inserted in the Article)

3. Have the authors made all data underlying the findings in their manuscript fully available?

The [PLOS Data policy](http://www.plosone.org/static/policies.action#sharing) requires authors to make all data underlying the findings described in their manuscript fully available without restriction, with rare exception (please refer to the Data Availability Statement in the manuscript PDF file). The data should be provided as part of the manuscript or its supporting information, or deposited to a public repository. For example, in addition to summary statistics, the data points behind means, medians and variance measures should be available. If there are restrictions on publicly sharing data—e.g. participant privacy or use of data from a third party—those must be specified.

Reviewer #1: No

Reviewer #2: No

Reviewer #3: Yes

**Answer**: This is a cross-sectional study, with a strictly qualitative approach. Considering that part of the interviews contain information that might identify the participants, the researchers committed themselves to treat the identity of the participants according to professional standards of confidentiality, since they contain particular elements and names of people and/or professionals. Thus, the investigation was based on the Brazilian norms that regulate research with human beings (resolution no. 466, of December 12, 2012, of the National Health Council/Ministry of Health) that uphold the confidentiality of the identification data of the research members, we chose not to provide the complete transcripts. However, speech clippings (transcriptions) were used as a way to represent the findings of the study, seeking to exemplify the categories listed from the content analysis applied.

4. Is the manuscript presented in an intelligible fashion and written in standard English?

PLOS ONE does not copyedit accepted manuscripts, so the language in submitted articles must be clear, correct, and unambiguous. Any typographical or grammatical errors should be corrected at revision, so please note any specific errors here.

Reviewer #1: Yes

Reviewer #2: Yes

Reviewer #3: Yes

**Answer**: Thank you!

5. Review Comments to the Author

Please use the space provided to explain your answers to the questions above. You may also include additional comments for the author, including concerns about dual publication, research ethics, or publication ethics. (Please upload your review as an attachment if it exceeds 20,000 characters)

Reviewer #1: This is an article about the perception of women with breast cancer during the pandemic. I congratulate the authors for the important initiative, but the impact in this context did not seem clear to me (pre-pandemic versus during the pandemic). A direct comparison between perceptions before and during the pandemic could shed light on this scenario. As a suggestion, objective questions that could be compared between periods could be asked with a larger number of patients.

**Answer**: Since this is an unexpected event, at the global level, COVID-19 interfered in the data collection of the study, with part of the collection performed during the pandemic due to the situation. However, this article was a cross-sectional study of a larger longitudinal research that is in the process of completion, and whose main focus is on a health education group intervention for self-care in breast cancer. Thus, a directly comparative analysis was not performed regarding the perceptions about the discovery of neoplasia in the pre-pandemic and pandemic periods.

Reviewer #2: A study on a very interesting aspect of perceiving cancer, in this case breast cancer, in the context of the COVID-19 pandemic. However, the study was conducted on too small a group to draw any more general conclusions. In my opinion, the questionnaire prepared in a vague way did not allow to draw any interesting conclusions. The results section lacks any statistical elaboration to draw more general conclusions regarding breast cancer detection delays during the pandemic. There is also no summary, in my opinion, of what aspects related to the treatment of breast cancer were the most difficult for patients. In the results section, answers from patient surveys are entered.

**Answer**: This is a cross-sectional study of a longitudinal research, of doctorate level, which is in progress, and had as its main objective the realization of an intervention in a group of health education for self-care in breast cancer, having as a sample women undergoing chemotherapy treatment. Since the pandemic interrupted data collection, there was a need to re-adjust the objectives and the intervention.

In this cross-sectional, descriptive and qualitative approach, we sought to understand the perceptions of women with breast cancer regarding the diagnosis. The sample of 40 participants, for a qualitative study, has been considered sufficient by other authors to achieve the understanding of an event such as this. It is understood that this approach has limitations regarding the generalization of the findings, which are restricted to the sample addressed in the article. Because breast cancer is a disease of importance in public health, although with limitations, it is intended to alert health professionals to have a broader look on the aspects that involve all the stages of the disease, ever since the discovery of a breast alteration, up until the emotional impact generated by the diagnosis and the possible implications of an unusual situation, as was the COVID-19 pandemic.

Since the interview script was elaborated and tested for further research, the questions addressed more in-depth questions about self-care during cancer treatments.

However, the question used to start the interview and dive into the theme of breast cancer raised interesting and profound reports on the discovery of the neoplasia, arousing the authors' interest in sharing such information.

Many women discovered the cancer by themselves, despite having mammographs done, even as often as the Ministry of Health in Brazil recommends for the control and screening of breast neoplasia. They also showed the impact and emotional repercussions of the diagnosis, such as the feelings of helplessness generated by the isolation brought by the pandemic.

In order to improve the quality of the article and comply with the reviewer's suggestions, some adaptations were made in the "Conclusion", which we hope has reached the expectations of the reviewer. It is noteworthy that, from the perspective of the qualitative approach, the interviewees' perceptions on the theme are considered, assuming the restrictions of this type of study with regard to the generalization and representativeness of the results for a broader population.

Considering the larger study, the analysis of the material explored (interviews) gave rise to several thematic axes and their categories, including the discovery of breast cancer, beliefs about the disease, perceptions regarding treatments and self-care during the treatment period.

Reviewer #3: Replace "middle" with "peak" in the topic. It may be relatively difficult to determine the middle of COVID-19 Pandemic.
220: Table 1 (Marital Status should broken into two. 1. a. Ever married b. Never married 2. If ever married, what is the current state of marriage? a. Currently with spouse b. Separated from couples c. Divorced d. Widow. Common law marriage should expunged. The information on the City as provided in the Table 1 should be removed but discussions around it should be in the research methodology. The mix of qualitative and quantitative data enriches this submission.

**Answer**: As suggested by the reviewer, the table was adapted with regard to the city of origin. It has also been corrected with regard to marital status, based on the classification of other studies published in this renowned journal [Della Vecchia C, Préau M, Carpentier C, Viprey M, Haesebaert J, Termoz A, Dima AL, Schott AM. Illness beliefs and emotional responses in mildly disabled stroke survivors: A qualitative study. PLoS One. 2019 Oct 23;14(10):e0223681. doi: 10.1371/journal.pone.0223681.]; [Serafim AP, Durães RSS, Rocca CCA, Gonçalves PD, Saffi F, Cappellozza A, Paulino M, Dumas-Diniz R, Brissos S, Brites R, Alho L, Lotufo-Neto F. Exploratory study on the psychological impact of COVID-19 on the general Population Brazilian. PLoS One. 2021 Feb 3;16(2):e0245868. doi: 10.1371/journal.pone.0245868. PMID: 33534820; PMCID: PMC7857630.]

This article is a presentation of a cross-sectional approach to a larger longitudinal research, with a main focus on a health education group intervention for self-care in breast cancer. Given the richness and depth of the results obtained in the interviews, several thematic axes emerged that will be explored in other articles.

As the research is still ongoing, we intended to perform an analysis with mixed methods, which should be presented shortly.

6. PLOS authors have the option to publish the peer review history of their article ([what does this mean?](https://journals.plos.org/plosone/s/editorial-and-peer-review-process#loc-peer-review-history)). If published, this will include your full peer review and any attached files.


If you choose “no”, your identity will remain anonymous but your review may still be made public.


**Do you want your identity to be public for this peer review?** For information about this choice, including consent withdrawal, please see our [Privacy Policy](https://www.plos.org/privacy-policy).

Reviewer #1: No

Reviewer #2: No

Reviewer #3: **Yes:** Professor Femi Rufus TINUOLA

**Answer**: We, the authors, really appreciate the attention and collaboration made for improvements in the research. Do not hesitate to contact us in case any further clarifications or changes are needed

.

[NOTE: If reviewer comments were submitted as an attachment file, they will be attached to this email and accessible via the submission site. Please log into your account, locate the manuscript record, and check for the action link "View Attachments". If this link does not appear, there are no attachment files.]

While revising your submission, please upload your figure files to the Preflight Analysis and Conversion Engine (PACE) digital diagnostic tool, <https://pacev2.apexcovantage.com/>. PACE helps ensure that figures meet PLOS requirements. To use PACE, you must first register as a user. Registration is free. Then, login and navigate to the UPLOAD tab, where you will find detailed instructions on how to use the tool. If you encounter any issues or have any questions when using PACE, please email PLOS at [figures@plos.org](mailto:figures@plos.org). Please note that Supporting Information files do not need this step.

*In compliance with data protection regulations, you may request that we remove your personal registration details at any time.* [*(Remove my information/details)*](https://www.editorialmanager.com/pone/login.asp?a=r)*. Please contact the publication office if you have any questions.*
